# Leukocyte Telomere Length and Neuregulin-4 Levels in Female Patients with Acromegaly: The Relationship between Disease Activity and Body Fat Distribution

**DOI:** 10.3390/jcm12124108

**Published:** 2023-06-17

**Authors:** Meric Coskun, Alev Eroglu Altinova, Afruz Babayeva, Aydin Tuncer Sel, Dilek Yapar, Mine Karaca, Mehmet Muhittin Yalcin, Mujde Akturk, Fusun Balos Toruner, Mehmet Ayhan Karakoc, Ilhan Yetkin

**Affiliations:** 1 Department of Endocrinology and Metabolism, Faculty of Medicine, Gazi University, Ankara 06100, Turkey; alevaltinova@yahoo.com (A.E.A.); dr.afruz87@gmail.com (A.B.); a_tuncersel@yahoo.com (A.T.S.); yalcin.muhittin@gmail.com (M.M.Y.); mujdeakturk@gmail.com (M.A.); fusunbalostoruner@yahoo.com (F.B.T.); ayhankarakoc@gazi.edu.tr (M.A.K.); ilhanyetkinster@gmail.com (I.Y.); 2 Department of Public Health, Faculty of Medicine, Gazi University, Ankara 06100, Turkey; dilekceliker@outlook.com; 3 Department of Internal Medicine, Faculty of Medicine, Gazi University, Ankara 06100, Turkey; minekaraca45@gmail.com

**Keywords:** acromegaly, leukocyte telomere length, neuregulin-4, fat distribution

## Abstract

The study aimed to examine leukocyte telomere length (LTL) and serum neuregulin-4 levels and their relationship with disease activity, co-morbidities and body fat distribution in female acromegaly patients. Forty female patients with acromegaly and thirty-nine age and body mass index (BMI) similar healthy female volunteers were included in the study. Patients were classified into two groups: active acromegaly (AA) and controlled acromegaly (CA). The quantitative polymerase chain reaction (PCR) method was used to study LTL, and T/S ratio < 1 was accepted as shortened telomere length. Neuregulin-4 was studied by ELISA. There was no difference in median LTL between acromegaly and the control group (*p* = 0.530). The percentage of T/S < 1 in patients with acromegaly (60.0%) was similar to that of the control group (43.6%) (*p* = 0.144). However, serum neuregulin-4 was significantly higher in patients with acromegaly than those in the control group (*p* = 0.037). There were no significant differences concerning LTL, percentage of T/S < 1 and neuregulin-4 levels between active and controlled acromegaly groups (*p* > 0.05). Neuregulin-4 correlated positively with fasting glucose, triglyceride (TG), triglyceride/glucose (TyG) index, and lean body mass in the acromegaly group. A negative correlation was observed between LTL and neuregulin-4 in the control group (*p* = 0.039). When the factors affecting neuregulin-4 were evaluated by multivariate linear regression analysis with an enter method, TG (β: 0.316, *p* = 0.025) was independently and positively associated with neuregulin-4. Our findings indicate that acromegaly is associated with unchanged LTL and high neuregulin-4 levels in female patients. However, the relationship between acromegaly, the aging process, and neuregulin-4 involves complex mechanisms, and further studies are needed.

## 1. Introduction

Acromegaly is a disease characterized by excessive growth hormone (GH) secretion and consequently increased secretion of insulin-like growth hormone (IGF-1), usually in a growth hormone-producing pituitary tumor [1]. Although the typical clinical feature is acral growth, co-morbidities such as diabetes mellitus (DM), hypertension, hyperlipidemia and cardiovascular disease may accompany it [2]. Epidemiological studies have shown that mortality is increased in patients with acromegaly, and this increase is significantly associated with cardiovascular, cerebrovascular and respiratory diseases [3]. Life expectancy in patients with acromegaly are affected by the biochemical control of IGF-1 [4].

Telomere is a nucleoprotein structure that covers the ends of chromosomes and maintains the stability of the genome [5]. Telomere length varies greatly between species and ranges from 10 to 15 kb in humans [6]. Telomere protects the chromosomal ends and provides proper chromosome replication [7]. Telomeres become progressively shorter with each cell division due to the end replication problem. This results in cellular senescence or apoptotic cell death [8]. Normal diploid cells lose telomeres with each cell cycle. Telomere length, therefore, decreases over time and may predict lifespan [9]. Leukocyte telomere length (LTL) is considered a marker for cellular senescence [10,11]. Factors affecting LTL include gender (longer in women), age, body mass index (BMI), chronic inflammation and chronic diseases [12,13,14]. IGF-1 is an essential regulator of cell growth and proliferation, and serum concentration decreases with age [15]. In healthy adults, a positive correlation was reported between age-adjusted LTL and IGF-1 levels [16].

As our knowledge increases, the property of brown adipose tissue (BAT) as an endocrine organ has become clearer [17]. Neuregulin-4 is a novel secreted batokine involved in the modulation of glucose, lipid metabolism and energy homeostasis [18]. Neuregulin-4, a member of the epidermal growth factor family, is expressed in lung, heart and adipose tissues with the highest in BAT [19]. Neuregulin-4 stimulates the browning of white adipose tissue when exposed to cold [20] and markedly increases during brown adipocyte differentiation [21]. Neuregulin-4 associated especially with insulin resistance, obesity and obesity-related metabolic dysregulations is a protective factor [18]. There is evidence that it is down-regulated in the presence of obesity [21]. In addition, it was found to be higher in women than in men and had a negative correlation with body fat mass [22]. In addition, neuregulin-4 targets the liver, reducing diet-induced insulin resistance and hepatic lipogenesis [23]. In experimental studies, in neuregulin-4 null mice, an increase in the amount of triglyceride (TG), development of hepatic steatosis and associated liver enzyme elevation were observed [21]. Protection from hepatic steatosis was also found in subjects with high neuregulin-4 levels [21,24]. A positive correlation has been shown between neuregulin-4 and body mass index (BMI), waist circumference, hip circumference and TG, which is negative in terms of high-density lipoprotein (HDL) cholesterol [25]. Increased GH and its treatment in acromegaly leads to prominent changes in visceral and subcutaneous fat mass and body composition [26]. In addition, insulin resistance, dysregulated glucose metabolism and dyslipidemia are common in acromegaly [27]. In light of this information, exploring the neuregulin-4 level, which is closely associated with the modulation of glucose and lipid metabolism, may be important regarding hormonal control and metabolic changes in patients with acromegaly.

LTL, which is an aging marker, is shortened by the effect of glucose metabolism disorder and excess body fat [28]. Neuregulin-4, a batokine released from brown adiposity, is associated with metabolic homeostasis and aging [29,30]. As far as we know, no study exists in the literature clarifying the relationship between neuregulin-4 and LTL. Only irisin, which is associated with browning of white adipose tissue, was found to be positively related to telomere length [31].

In this study, we aimed to investigate LTL and neuregulin-4 levels in female patients with acromegaly and compare them with healthy controls. We also planned to examine the association of LTL and neuregulin-4 with body fat distribution and other metabolic parameters.

## 2. Materials and Methods

### 2.1. Case Selection

In the literature, it has been observed that both telomere length and neuregulin-4 are affected by gender, and they are higher in women than in men [13,22,32]. Therefore, the study was planned to include only female patients with acromegaly so that the gender difference would not affect the results. Forty women with acromegaly followed in our endocrinology and metabolism clinic were included in the study. Acromegaly was diagnosed by failure of suppression of serum GH concentrations below 1 ng/mL after a 75 g oral glucose tolerance test (OGTT) together with fasting serum IGF-1 concentrations above the normal ranges for age and gender with the presence of clinical features of acromegaly [2]. Transsphenoidal surgery was performed as the first treatment option in all patients. Gamma-knife radiosurgery was performed in patients when needed. Those with the upper limit of normal (ULN) <1.2 for IGF-1 at three months after surgery or under medical treatment were accepted as controlled acromegaly, and those without as active acromegaly [33].

Demographic data of the patients, duration of the disease, comorbid diseases, surgery, drug use, radiosurgery data and the smoking status of the cases were noted. Exclusion criteria were as follows: younger than 18 years of age; untreated hormonal deficiencies, uncontrolled HT, DM and coexisting active inflammatory and infectious diseases. None of our patients had hormonal co-production. Twelve (30%) of the acromegaly patients were newly diagnosed. Twenty-eight patients (70%) were previously diagnosed and underwent transsphenoidal surgery. The median follow-up period of these patients was nine (3–25) years. Fifteen patients (37.5%) were followed up with medical treatment after surgery. One patient was treated with cabergoline, four patients with lanreotide, four patients with octreotide, three patients with octreotide and cabergoline, one patient with lanreotide and cabergoline, and two patients with octreotide and pegvisomant. Cases were divided into two groups according to disease activity. In total, 17 (42.5%) of the acromegaly patients were included in the active acromegaly (AA) group and 23 (57.5%) in the controlled acromegaly (CA) group. The CA group consisted of patients who were in remission for at least six months. Eight (20%) patients with acromegaly had hypertension, fifteen (37.5%) DM, and eight (20%) hypothyroidism under proper treatment. Nine patients with DM used metformin; six patients used metformin and basal insulin. Since telomere length is affected by age, BMI and smoking, apart from gender, healthy controls were selected from cases who came to the outpatient clinic for routine control with similar gender, age, BMI, and smoking status [12,34].

### 2.2. Ethical Approval

The study was approved by the local ethics committee (22.11.2020-791). Written informed consent was obtained from all subjects involved in the study.

### 2.3. Body Composition Analysis

Body composition analysis was performed with the bioelectrical impedance analysis (BIA) Tanita BC-418 MA Body Composition Analyzer (Tanita Corporation, Tokyo, Japan), a fast, low-cost and easy measurement method. Total fat percentage and mass and lean body mass were measured. In addition, ViScan AB-140M Abdominal Fat Analyzer (Tanita Corporation, Tokyo, Japan) was used to measure visceral and truncal fat.

### 2.4. Laboratory Measurements

Serum samples for analyses were obtained early in the morning after overnight fasting. Serum fasting glucose, total serum cholesterol (TChol), HDL cholesterol, low-density lipoprotein (LDL) cholesterol, TG levels and high sensitivity CRP were measured in patients and controls. Triglyceride/glucose (TyG) index was calculated with the formula Ln (TGxGlucose)/2. GH and IGF-1 levels were recorded when enrollment of the acromegaly cases and ULN (patient’s IGF-1 level/reference IGF-1 upper limit according to age and gender) was calculated to differentiate between active and controlled acromegaly.

For neuregulin-4 measurement, patient and control samples were centrifuged and stored at −80 °C until the day of analysis. Before analysis, serum samples were first transferred to −20 °C and then to room temperature. MyBioSource human neuregulin-4 ELISA (catalog no: MBS2024409, detection range 0.156–10 ng/mL, sensitivity < 0.056 ng/mL) was used. The intra-assay coefficient of variation (CV) was less than 10%, and the inter-assay CV was less than 12%.

Total blood samples in EDTA tubes were stored at −20 °C after sampling. Freeze-thaw was performed once before the PCR reaction. DNA extraction was performed with the QuickGene DNA Extraction Whole Blood Kit S (Kurabo, Neuss, Germany) in accordance with the protocol. DNA samples were stored at −20 °C. DNA quality was measured with the Colibri Microvolume Spectrometer (Titertek-Berthold, Germany). DNA quantity and quality were evaluated with OD260/OD280 and OD260/OD230 ratios. Relative LTL was measured in all groups using the quantitative polymerase chain reaction (qPCR) method. A Biorad CFX96 Real Time PCR device was used in the analysis. ScienCell’s Absolute Human Telomere Length Quantification qPCR Assay Kit (catalog no: 8918, AHTLQ, USA) was designed to directly measure the average telomere length of a human cell population. The telomere primer set recognizes and amplifies telomere sequences. The single-copy reference (SCR) primer set recognizes and strengthens a 100 bp-long region on human chromosome 17 and serves as a reference for data normalization. Each primer set has been validated by qPCR with melt curve analysis and gel electrophoresis for amplification specificity and by template serial dilution for amplification efficiency. Telomere length was expressed as a relative T/S ratio calculated as the ratio of telomere repeat copy number (T) to single gene copy reference (S) for each sample using the formula 2ΔCt(sample)/2ΔCt(Calibrator) = 2^−ΔΔCt^ [35,36]. We designated the control group as the calibrator, and the relative telomere length of each sample was determined accordingly. As in the previous studies, we considered the T/S ratio < 1 in patients and controls to have short telomere lengths [35].

### 2.5. Statistical Analysis

All analyzes were performed using commercial statistical software (version 22.0; IBM SPSS). Continuous variables were analyzed for homogeneity of variance using the Kolmogorov–Smirnov test. Continuous variables were presented as the mean ± SD or median (minimum–maximum). Normally distributed data were analyzed with the t-test, and unevenly distributed data were analyzed with the Mann–Whitney U test. The Chi-square test was used in the analysis of categorical data. We performed a Spearman’s Rho correlation analysis between LTL, serum neuregulin-4, hs-CRP, fasting glucose, creatinine, IGF-1, GH, lipid levels and body fat assays. To better explain the causality relationship, multivariate linear regression analysis with an enter method was performed and log transformation was applied for neuregulin-4, TG and fasting glucose, which showed non-parametric distribution. We considered *p* values less than 0.05 to be statistically significant.

## 3. Results

Demographic, clinical and laboratory parameters of acromegaly and control subjects are shown in Table 1. There was no significant difference between the two groups regarding age and smoking (*p* > 0.05). While BMI, total body fat, visceral fat and truncal fat were similar between acromegaly and control groups. The percentage of lean body mass was similar between acromegaly and control groups and lean body mass (kg) (*p* < 0.001) was higher in the acromegaly group than in the control group. Fasting glucose (*p* < 0.001) TG levels (*p* < 0.001) were significantly higher and HDL was lower (*p* = 0.032) in the acromegaly group than in the control group. TyG index was significantly higher in the acromegaly group than in the control group (4.72 (4.37–5.76) vs. 4.47 (4.03–4.99), *p* < 0.001). There was no significant difference (813.2 (459.2–2062) vs. 876.6 (486.8–2422) ng/mL, *p* = 0.224) in terms of hs-CRP between the groups.

**Figure 1:**
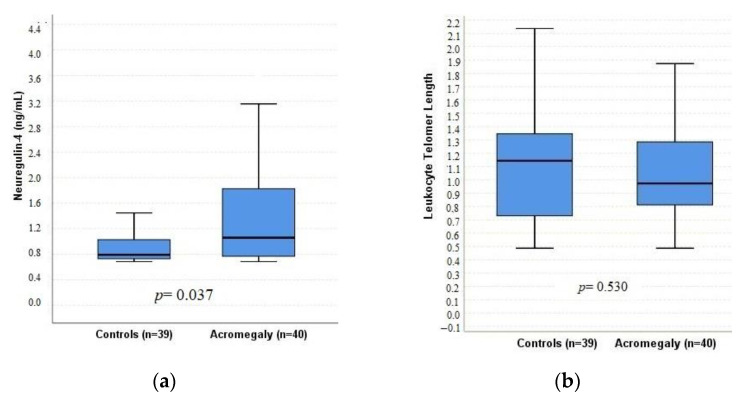
Box plots of neuregulin-4 (**a**) and LTL (**b**) values for the control and acromegaly groups.

As shown in Table 1, although the median LTL was shorter in the acromegaly group than the control group, this difference was not statistically significant (0.973 vs. 1.14, *p* = 0.530). The percentage of having short telomere length (T/S < 1) was also similar between the groups (60% vs. 43.6%, *p* = 0.144). However, median neuregulin-4 levels were significantly higher in the acromegaly group than the control group (1.06 vs. 0.79 ng/mL, *p* = 0.037) (Figure 1).

**Figure 2:**
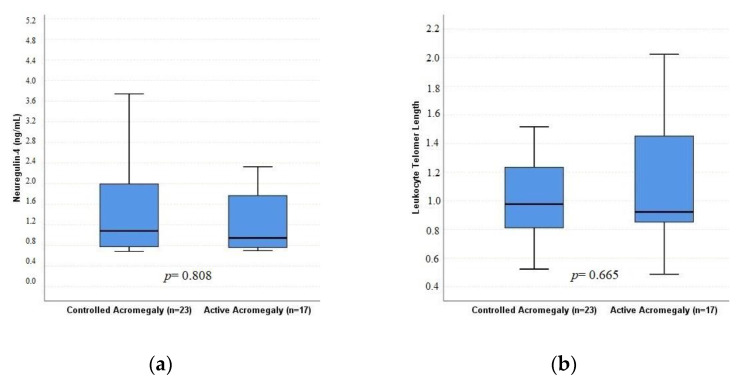
Box plots of neuregulin-4 (**a**) and LTL (**b**) values for the controlled acromegaly (CA) and active acromegaly (AA) groups.

The AA and CA groups were similar in terms of age, presence of DM, presence of hypertension and use of medical therapy. In addition, BMI, lean body mass (kg), visceral fat and truncal fat were similar between groups (*p* > 0.05). In contrast, fat percentage (35.35 vs. 29.73, *p* = 0.023) and fat mass (27.83 vs. 21.9, *p* = 0.046) were higher, while lean body mass percentage was lower (64.45 vs. 69.80 *p* = 0.028) in the CA group than in the AA group. Fasting glucose, creatinine, lipid parameters and TyG index were similar between the groups (*p* > 0.05). As shown in Figure 2, while LTL and neuregulin-4 were similar in the AA and CA groups, hs-CRP was significantly lower in the AA group than in the CA group (630.2 vs. 876.6 ng/mL, *p* = 0.045) (Table 1).

Twenty-eight patients previously diagnosed with a median follow-up of nine (3–25) years were divided into two groups, less than ten years and ten years and above, to evaluate the effect of duration of the disease on LTL. Twelve (42.9%) were followed for ten years or more, and sixteen (57.1%) were followed for less than ten years. No statistically significant results were found between the two groups in terms of LTL (1.12 (0.71–1.35) vs. 0.87 (0.52–2.02) *p* = 0.725) and the percentage of having short telomere length (T/S < 1) (41.7% vs. 75% *p* = 0.074). When the treated and untreated patients were compared, no difference was found between the groups regarding BMI, lean body mass, glucose, TG, neuregulin-4 and LTL (*p* > 0.05).

**Figure 3:**
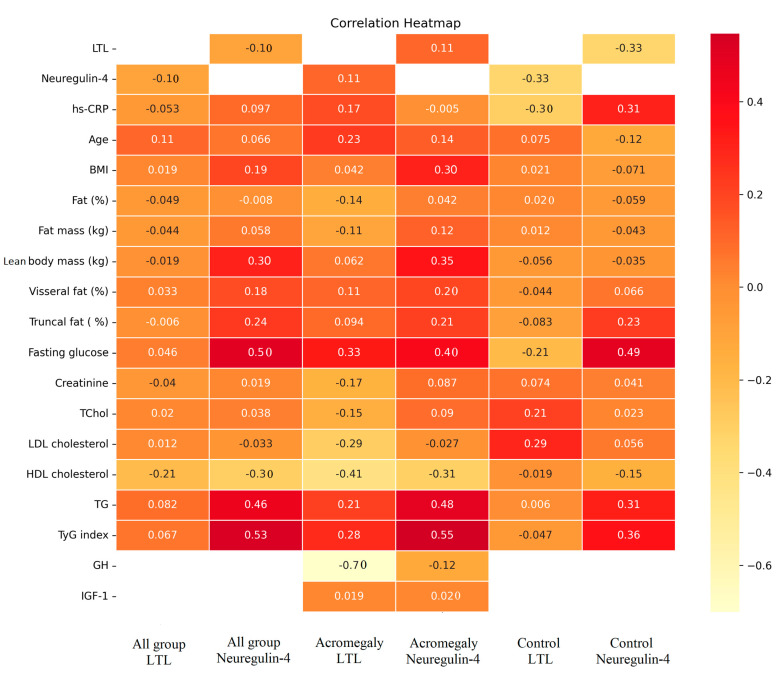
Correlation heatmap of LTL and neuregulin-4 levels with metabolic, anthropometric and laboratory parameters. In the correlation heatmap, r-value, which is ≤ 0.10, is given with three decimals and > 0.10 with two decimals. LTL: leukocyte telomer length, BMI: body mass index, GH: growth hormone, IGF-1: insulin-like growth factor-1, TChol: total cholesterol, TG: triglyceride, HDL: high-density lipoprotein cholesterol, LDL: low-density lipoprotein cholesterol, hs-CRP: high sensitivity-C reactive protein, TyG index: triglyceride/glucose index.

The correlation analyses of LTL and neuregulin-4 with metabolic, anthropometric and laboratory parameters is shown in Figure 3. LTL was not associated with any parameter in the whole group analysis (*p* > 0.05). Considering the separate group analyses, LTL was negatively associated with neuregulin-4 (*p* = 0.039) in the control group, but no correlation was found with neuregulin-4 in the acromegaly group (*p* > 0.05). In the acromegaly group, LTL was observed to be associated with fasting glucose (*p* = 0.037) and HDL (*p* = 0.008) levels.

In the whole group analysis, neuregulin-4 had a positive correlation with lean body mass (*p* = 0.009), truncal fat (*p* = 0.039), fasting glucose (*p* < 0.001), TG (*p* < 0.001) and TyG index (*p* < 0.001). In addition, there was a negative correlation between neuregulin-4 and HDL cholesterol (*p* = 0.008). In the acromegaly group, lean body mass (r = 0.347, *p* = 0.033), fasting glucose (r = 0.402, *p* = 0.010), TG (r = 0.480, *p* = 0.002) and TyG index (r = 0.547, *p* < 0.001) were positively associated with neuregulin-4.

A linear regression analysis model with an enter method, including BMI, age, lean body mass, HDL cholesterol, fasting glucose and TG was created to assess the factors affecting neuregulin-4. As shown in Table 2, when the factors affecting neuregulin-4 were evaluated by multivariate linear regression analysis, TG (β: 0.316, *p* = 0.025) independently and positively was associated with neuregulin-4.

## 4. Discussion

In the present study, neuregulin-4 levels were found to be higher in female patients with acromegaly than controls. As far as we know, there is no study regarding this relationship between acromegaly and neuregulin-4 in the literature. In addition, LTL was found to be similar between acromegaly and control groups, and LTL was not affected by disease activity.

GH and IGF-1 have essential roles in the regulation of metabolism and body composition [37]. GH is lipolytic and an important regulator of fat mass and it induces insulin resistance in the liver and muscle at supraphysiological levels [38]. In acromegaly, changes in body fat distribution have been reported previously, indicating decreased visceral and subcutaneous adipose tissue and increased intramuscular adipose tissue [39]. As the cause of increase in circulating neuregulin-4 in acromegaly, one can speculate that increased ectopic intramuscular adipose tissue may be the source of neuregulin-4. On the other hand, this increase of neuregulin-4 might be a preventive mechanism for insulin resistance caused by the effect of GH. As mentioned before, neuregulin-4 null mice have high TG levels. Neuregulin-4 activates ErbB3 and ErbB4, which are the epidermal growth factor family of receptor tyrosine kinase signaling in hepatocytes; neuregulin-4 also negatively regulates de novo lipogenesis [21]. Supporting these results, transgenic activation of neuregulin-4 signaling results in increased whole-body glucose turnover and glycolysis, partly due to increased skeletal muscle glucose utilization. Furthermore, hepatic fatty acid β-oxidation and ketogenesis were identified as novel downstream targets of neuregulin-4 in hepatocytes [24]. Taken together, an increase in neuregulin-4, a batokine responsible for the regulation of glucose, lipid and energy homeostasis, might be a reactive response to increased glucose and lipid levels in acromegaly.

Increased growth hormone and IGF-1 in acromegaly generally cause changes in body composition with anabolic, lipolytic and sodium retention effects. Many studies have shown that lean body mass and body water increase and body fat decreases in active acromegaly. After biochemical control is achieved, largely extracellular fluid decreases, body fat increases, and lean body mass decreases [40,41]. In our study, while body fat levels were similar between the acromegaly and control groups, the lean body mass (kg) was higher in the acromegaly group. When the patients were divided in terms of disease activity, body fat mass and percentage were higher, and the rate of lean body mass was lower in the CA group compared to the AA group. In addition, Gil et al. have stated that the treatment of acromegaly impacts body composition [42]. In our study, when the patient group was divided into two groups in terms of medical treatment, body composition, metabolic parameters, LTL and neuregulin-4 were found to be similar between the groups.

Neuregulin-4 was positively correlated with lean body mass in the acromegaly group in our study. A positive correlation has been found between neuregulin-4 and lean body mass in a previous study covering obese men [43], similar to our result. Lean body mass and neuregulin-4 levels were significantly higher in our acromegaly group than in the control group. Therefore, a question may arise that elevated neuregulin-4 levels may not result from acromegaly directly, but it is difficult to separate them. Nevertheless, multivariate linear regression analysis failed to show the effect of lean body mass on neuregulin-4. Taken together, the association between neuregulin-4 and body fat distribution in acromegaly deserves further research.

In the whole group analysis, there was positive correlation between neuregulin-4, fasting glucose and TG levels, and negative correlation with HDL cholesterol. In the separate analysis, both acromegaly and control groups showed a positive correlation between neuregulin-4 and fasting glucose. A positive correlation was observed between neuregulin-4 and TG in the acromegaly group, similar to the whole group analysis. TyG index was correlated, which has been proposed as a strong indicator of insulin resistance [44], and in our study, neuregulin-4 showed a significant positive correlation with TyG index in both whole group and separated group analyses. Consistent with our findings, a positive correlation between neuregulin-4, fasting glucose and TG, and a negative correlation with HDL cholesterol have been reported in type 2 diabetes, highlighting the increase of neuregulin-4 in the presence of impaired metabolic control [25,45]. Hyperglycemia as a result of insulin resistance often coexists in patients with acromegaly. Considering the reported associations between neuregulin-4 and glucose metabolism and the positive correlation with fasting glucose found in the present study, it is difficult to say that increased neuregulin-4 level in the acromegaly group is due to the acromegaly itself. Moreover, neuregulin-4 was similar between CA and AA groups in our study. In addition, fasting glucose, lipid parameters and TyG index were also similar between the groups in terms of disease activity. Since the metabolic parameters were similar between the groups, it is an expected result that neuregulin-4 was similar between the groups.

On the other hand, in different studies examining neuregulin-4 in metabolic syndrome and non-alcoholic fatty liver disease, it has been shown that there is a negative correlation between neuregulin-4, fasting glucose and TG and a positive correlation with HDL cholesterol [22,46,47]. Despite detailed studies describing the response of neuregulin-4 to metabolic dysregulation at the molecular level [21,24], it is noteworthy that there are controversial results between neuregulin-4 and metabolic parameters in cross-sectional studies in the literature. Liu and Chen recently emphasized that neuregulin-4 has positive metabolic effects by increasing the sympathetic innervation of adipose tissue. They also pointed out that comprehensive evaluations are needed to explain the inconsistent results regarding neuregulin-4 levels in clinical studies [48]. This situation may be related to the complex receptor structure of neuregulin-4 [49,50]. Perhaps the resistance or excessive activation of the neuregulin-4 receptor level creates different neuregulin-4 responses to the deterioration in metabolic parameters. Zhang et al. found that neuregulin-4 receptor, ErbB4, is highly expressed in the hypothalamus and decreased phosphorylation of hypothalamic ErbB4 in diet-induced obese mice. Peripheral neuregulin-4 stimulated neurons in the paraventricular nucleus of the hypothalamus via ErbB4 while overexpression of ErbB4 in the paraventricular nucleus has a protective effect against obesity [51], which supports our current hypothesis.

It is known that there is a decrease in telomere length with aging [13]. IGF-1 also decreases with increasing age [52]. It has been observed that IGF-1 and LTL show a positive correlation with each other [16,53]. The lower normal range of IGF-1 in healthy individuals has been demonstrated to be associated with poor metabolic health and is considered a risk factor for mortality [54,55]. With respect to telomere length in the presence of high IGF-1, to the best of our knowledge, there is only one article in the literature. In that study, shorter telomere length was found in patients with acromegaly than the control patients with non-functional pituitary adenoma, who were compatible in terms of age, gender and DM, and telomere length in acromegaly showed a negative correlation with the duration of the disease. In vivo analyses of human skin fibroblasts found that IGF-1 rather than GH may cause telomere shortening, but the mechanism has not been clarified [56]. However, higher IGF-1 levels in the normal range have also been demonstrated to be related with higher LTL in older men [53]. Therefore, telomere shortening is an unexpected result in the presence of excess IGF-1. In our study, there was no statistically significant change in LTL in acromegaly, which is more consistent with the existing data.

The mean age at diagnosis of acromegaly ranges from 40 to 50 years, and the average delay in the diagnosis of acromegaly appears to be about 5.5 years [57,58]. Although the delay in diagnosis has decreased over the years, it is still a critical problem, and the effects of high IGF-1 exposure are observed even at the time of diagnosis [59,60]. Thus, the duration of the disease, which is quite long, may contribute to telomere length other than age. LTL and the percentage of having short telomere length were observed to be similar between the groups in our study. Twenty-eight of the patients with acromegaly included in the study were previously diagnosed, and almost half of these patients have been followed for over ten years. Normal GH values in the study belong to the controlled acromegaly group. Our study’s high percentage of controlled acromegaly might have eliminated the effect of the disease on LTL during the long follow-up period. Further analysis and exploration of LTL and also neuregulin-4 are certainly required with larger patient numbers with active disease and significantly worse metabolic control.

Some growth factors have been proposed to be associated with telomere length [61]. In our study, we found a negative correlation between LTL and neuregulin-4, which is a peptide from the growth factor family, in the control group, but not in the acromegaly group. We think that co-morbid conditions, which are frequently seen in acromegaly, may have a confounding effect on this association between LTL and neuregulin-4.

Regarding the limitations of our study, one is the lack of certain cut-off for short telomere length since different studies choose different values as cut-offs including <1 or tenth and fifth percentiles of the disease-free group [36,62]. The second is that our patients were not newly diagnosed and were taking some medications related to their co-morbidities. In our study, only female patients were included to eliminate the gender effect, which is a valuable feature of our work.

## 5. Conclusions

Serum neuregulin-4 levels are higher in female patients with acromegaly and appear to be associated with some metabolic components, suggesting that it might be a protective factor in acromegaly. However, it seems that acromegaly and disease activity have no considerable effects on LTL at least in female patients. On the other hand, the relationship observed between neuregulin-4 and LTL in healthy subjects needs confirmation with larger population studies.

## Figures and Tables

**Table 1:** Demographic, clinical and laboratory data of the acromegaly and control groups.

	Acromegaly (*n* = 40)	Controls (*n* = 39)	*p*	AA (*n* = 17)	CA (*n* = 23)	*p*
Age (year)	46.10 ± 8.79	42.82 ± 6.99	0.071	44.7 ± 9.88	47.13 ± 7.96	0.396
BMI (kg/m^2^)	29.61 ± 5.63	27.40 ± 4.18	0.520	28.06 ± 4.94	30.76 ± 5.93	0.136
Fat (%)	32.99 ± 7.61	34.99 ± 5.85	0.203	29.73 ± 8.88	35.35 ± 5.64	0.023
Fat mass (kg)	25.34 ± 9.07	25.21 ± 7.86	0.945	21.92 ± 9.06	27.83 ± 8.42	0.046
Lean body mass (kg)	49.56 ± 5.58	45.21 ± 3.80	<0.001	49.67 ± 5.30	49.47 ± 5.72	0.914
Lean body mass (%)	66.70 ± 7.50	65.08 ± 5.84	0.296	69.80 ± 8.69	64.45 ± 5.70	0.028
Visceral fat (%)	10.28 ± 4.36	9.01 ± 3.55	0.166	8.68 ± 4.37	11.36 ± 4.37	0.075
Truncal fat (%)	40.41 ± 8.90	38.74 ± 6.88	0.362	37.41 ± 11.44	41.16 ± 10.47	0.075
Fasting glucose (mg/dL)	104.5 (78–388)	88 (54–100)	<0.001	105 (75–338)	104 (74–270)	0.787
Creatinine (mg/dL)	0.61 ± 0.11	0.63 ± 0.08	0.354	0.61 ± 0.12	0.61 ± 0.10	0.909
GH (ng/mL)	1.17 (0.05–62.50)	-	-	3.74 (1.01–62.5)	0.58 (0.05–5.36)	<0.001
IGF-1 (ng/mL)	199 (46–704)	-	-	410 (240–704)	142 (46–241)	<0.001
TChol (mg/dL)	217.07 ± 51.72	219.74 ± 41.77	0.802	210.41 ± 55.33	222.00 ± 49.56	0.491
LDL cholesterol (mg/dL)	132.85 ± 40.07	141.00 ± 33.59	0.331	127.35 ± 43.41	136.81 ± 37.88	0.463
HDL cholesterol (mg/dL)	53.30 ± 10.54	58.71 ± 11.47	0.032	52.94 ± 11.56	53.56 ± 9.99	0.856
TG (mg/dL)	139 (69–387)	91 (44–232)	<0.001	145 (69–359)	138 (75–387)	0.850
TyG index	4.72 (4.37–5.76)	4.47 (4.03–4.99)	<0.001	4.83 ± 0.33	4.82 ± 0.33	0.887
hs-CRP (ng/mL)	813.2 (459.2–2062)	876.6 (486.8–2422)	0.224	630.2 (459.2–1868.0)	876.6 (459.2–2062)	0.045
LTL	0.973 (0.48–2.02)	1.14 (0.48–2.28)	0.530	0.92 (0.48–2.02)	0.97 (0.52–1.87)	0.665
T/S < 1 (%)	60	43	0.144	52.9	65.2	0.522
Neuregulin-4 (ng/mL)	1.06 (0.68–4.81)	0.79 (0.68–3.17)	0.037	0.94 (0.70–3.62)	1.08 (0.68–4.81)	0.808

AA: active acromegaly, CA: controlled acromegaly, BMI: body mass index, GH: growth hormone, IGF-1: insulin-like growth factor-1, TChol: total cholesterol, TG: triglyceride, TyG index: triglyceride/glucose index, HDL: high-density lipoprotein-cholesterol, LDL: low-density lipoprotein-cholesterol, hs-CRP: high sensitivity-C reactive protein, LTL: leukocyte telomere length. Twelve (30%) of the acromegaly patients were newly diagnosed. Twenty-eight patients (70%) were previously diagnosed and underwent transsphenoidal surgery. Biochemical control could not be achieved in five of these patients. Cases were divided into two groups according to disease activity, and 17 (42.5%) of the acromegaly patients were included in the AA group and 23 (57.5%) in the CA group. The CA group consisted of patients who were in remission for at least six months.

**Table 2:** Multivariate linear regression analysis between neuregulin-4 and age, BMI, lean body mass, fasting glucose, TG and HDL cholesterol.

	Beta Coefficients	*p*
BMI	−0.085	0.543
Age	−0.047	0.696
Lean body mass	0.105	0.465
HDL cholesterol	−0.128	0.291
Log fasting glucose	0.108	0.412
Log TG	0.316	0.025

Dependent variable: Log neuregulin-4, BMI: body mass index, TG: triglyceride, HDL: high-density lipoprotein cholesterol.

## Data Availability

The data used to support the findings of this research are available from the corresponding author upon request.
